# The Application of Autonomous Dental Implant Robots in Avoiding Maxillary Sinus and Making Full Use of Sinus Septa for Fixed Restoration Supported by Implants: A Case Report

**DOI:** 10.1155/crid/5072934

**Published:** 2026-10-05

**Authors:** Rui Xie, Yue Feng, Jianhong Duan, Peng Zhu, Xiaonan Yu, Yimin Zhao, Zhihong Feng

**Affiliations:** ^1^ Digital Dentistry Center, School of Stomatology, The Fourth Military Medical University, Xi′an, Shaanxi, China, fmmu.edu.cn; ^2^ State Key Laboratory of Oral & Maxillofacial Reconstruction and Regeneration, Xi′an, Shaanxi, China; ^3^ National Clinical Research Center for Oral Diseases, Xi′an, Shaanxi, China; ^4^ Shaanxi Key Laboratory of Stomatology, Xi′an, Shaanxi, China; ^5^ Department of Prosthodontics, School of Stomatology, The Fourth Military Medical University, Xi′an, Shaanxi, China, fmmu.edu.cn

**Keywords:** digital technology, implant restoration, maxillary sinus, tilted implantation

## Abstract

Implant rehabilitation in the atrophic posterior maxilla presents significant challenges, primarily due to pneumatized maxillary sinuses and the presence of septa. Although sinus floor elevation is a standard approach, utilizing native basal bone through tilted implant placement offers a less invasive alternative. However, avoiding the sinus cavity while anchoring implants in restricted bone volumes, such as sinus septa, is technically demanding with freehand methods. This case report describes the novel application of an autonomous dental implant robot to address these anatomical complexities. We present the case of a 65‐year‐old female requiring restoration for missing maxillary teeth 15–17. Radiographic evaluation revealed insufficient vertical bone height but identified a favorable maxillary sinus septum at Site 15 and adequate tuberosity bone at Site 17. To maximize native bone contact and avoid sinus perforation, two Straumann implants were planned and placed using an autonomous robotic system. Postoperative cone‐beam computed tomography confirmed high precision in implant positioning relative to the preoperative plan. At 15‐month follow‐up, the implants demonstrated osseointegration with no biological or mechanical complications, restoring satisfactory function and esthetics. This case illustrates that autonomous robotic assistance can significantly enhance surgical accuracy, enabling the safe and effective utilization of complex anatomical structures like sinus septa, thereby broadening treatment modalities for the compromised posterior maxilla.

## 1. Introduction

The widespread application of the tilted implant placement technique has solved many clinical problems and achieved favorable outcomes [1]. For example, in cases of insufficient bone volume in the posterior region, tilted implants make possible edentulous jaw implant restoration and allow for fixed prosthetic restoration of the posterior maxilla without the need for sinus floor elevation, thereby avoiding a series of issues associated with vertical and/or horizontal bone augmentation. When impacted teeth or large blood vessels are present in the edentulous area, tilted implants can also be used to bypass such structures for implant restoration [2, 3]. However, achieving the dual objectives of avoiding critical anatomical structures while ensuring optimal implant position and angulation to attain ideal prosthetic outcomes remains the ultimate goal during tilted implant placement. The application of digital technology in implant dentistry offers a reliable guarantee to achieve this goal. This case demonstrates, via the use of an autonomous implant robot, the excellent performance of digital technology in facilitating tilted implant placement to avoid the maxillary sinus and in utilizing the relatively wide and thick alveolar bone at the base of the sinus septum. This provides patients with an efficient implant treatment strategy characterized by minimal trauma, low risk, short duration, and reduced cost.

## 2. Case Presentation

The patient was a 65‐year‐old female who had several teeth extracted due to root fractures. In December 2023, she presented to the Department of Prosthodontics at the Third Affiliated Hospital of Air Force Medical University, seeking implant‐supported fixed prosthetic treatment. The patient had no history of systemic diseases, drug allergies, or any special medication use. Intraoral examination revealed the absence of Teeth 15, 16, and 17, with well‐healed extraction sockets, adequate keratinized gingiva, and normal intermaxillary distance. The residual alveolar ridge width at the edentulous sites was acceptable (Figure 1). Radiographic examination indicated the presence of a septum within the maxillary sinus at the site of Tooth 15, with the septal base approximately 10 mm from the crest of the alveolar bone. The floor of the sinus near the mesial and distal aspects of the septum arched toward the alveolar crest (Figure 2). At Sites 16 and 17, there was significant insufficiency in the sinus‐crest distance, and diffuse mucosal thickening within the sinus was observed. The bone volume in the maxillary tuberosity region was sufficient, with a usable bone width of approximately 7–8 mm when measured along a distal‐tilted axis, and the available bone height exceeded 15 mm (Figure 3).

**Figure 1:**
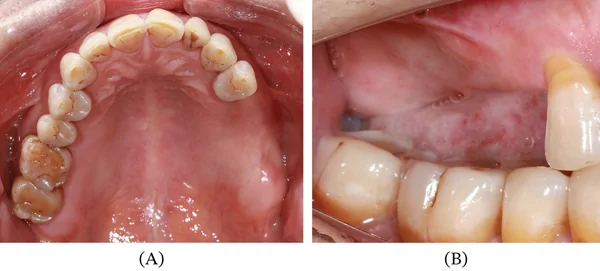
Pretreatment intraoral images. (A) Occlusal view. (B) Buccal view.

**Figure 2:**
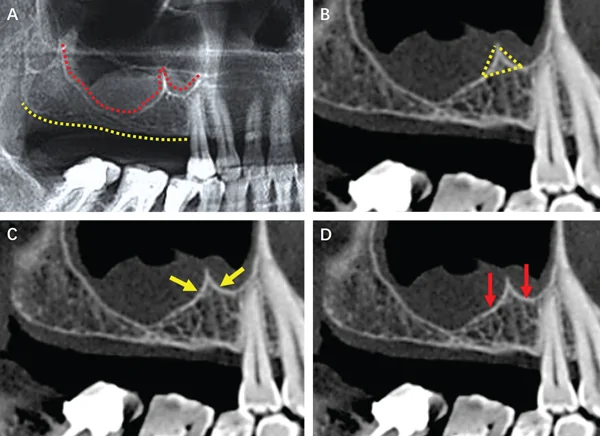
Bone volume and anatomical analysis at the implant site of 15. (A) The contour of the maxillary sinus floor is indicated by the red dashed line, and the contour of the alveolar crest is indicated by the yellow dashed line. A sinus septum is present at the site planned for implant placement at 15. (B) The alveolar bone at the base of the septum is indicated by the yellow dashed triangle. (C) The alveolar bone at the base of the septum is relatively wide and thick, as indicated by the yellow arrow. (D) The maxillary sinus floor at the mesial and distal aspects of the septum is positioned closer to the alveolar crest compared with the base of the septum, as indicated by the red arrows, resulting in a reduction of available bone volume for implantation.

**Figure 3:**
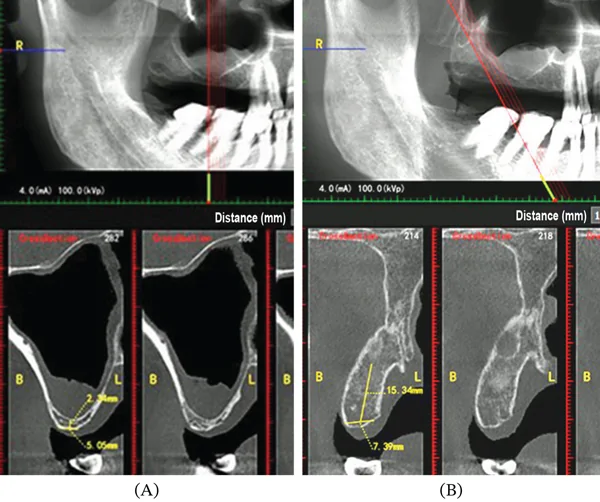
Analysis of bone volume and anatomical structures at the 16 and 17 implant sites and the maxillary tuberosity. (A) An evaluation for conventional axial implant placement at Sites 16 and 17 revealed that both the available bone height and width were significantly insufficient, and there is diffuse thickening of the sinus mucosa. (B) The bone volume in the maxillary tuberosity region is sufficient to meet the requirements for placing a tilted implant at 17.

According to the principles of prosthetically driven implant placement, it was planned to insert implants at Sites 15 and 17, with their positions, angulations, and dimensions meeting the requirements for a fixed prosthetic restoration. For the implant at Site 15, the wider and higher available bone at the base of the maxillary sinus septum would be fully utilized, placing the apical end of the implant directly against the septal base. The sinus floor at both the mesial and distal sides of the septum is lower than at the septal site, so the apical end of the implant should be prevented from deviating toward the mesial or distal areas adjacent to the septum; implant placement toward these lower sinus floor regions should be avoided. For the implant at Site 17, the abundant available bone in the maxillary tuberosity region would be utilized by tilting the apical end of the implant distally, placing it along the posterior wall of the maxillary sinus, with an inclination not exceeding 45°. Preoperative preparation of the autonomous implant robot was performed in accordance with the treatment plan, including the design and 3D printing of a positioning guide (Figure 4). The autonomous dental implant robotic system used in this case was the commercially available YakeBot oral surgical navigation and positioning system/autonomous dental implant robotic system. The system included a vision cart with an optical tracking unit, a robotic arm, an electric motor and implant handpiece, a dental implant surgical positioning assembly, a surgical positioning guide, a wireless touch screen, and dedicated software for patient data management, preoperative planning, intraoperative navigation, robotic control, and postoperative evaluation. Preoperative CBCT data were imported in DICOM 3.0 format, with a slice thickness of ≤ 1 mm and an in‐plane resolution of at least 512 × 512 pixels, according to the manufacturer′s requirements. The software was used for three‐dimensional reconstruction, panoramic curve generation, prosthetically driven implant planning, positioning guide design, and intraoperative navigation.

**Figure 4:**
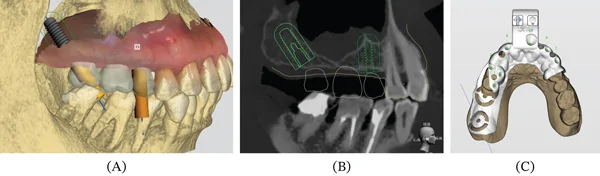
Treatment planning and preoperative preparation for autonomous implant robotic surgery. (A) Planning of implant positions and angulations according to the requirements of the prostheses. (B) Implant design utilizing both the base of the sinus septum and the maxillary tuberosity. (C) 3D printing of the positioning guide.

During surgery, the optical tracking unit mounted on the vision cart monitored the spatial relationship among the patient, the positioning guide, the robotic arm/end effector, and the implant handpiece. Before osteotomy, the positioning guide was placed, the dental implant surgical locator components were connected, and patient registration, robotic arm/end‐effector calibration, handpiece calibration, and verification of the planned entry point and drilling trajectory were completed according to the software‐guided manufacturer′s protocol. The operator interacted with the system through the graphical user interface and wireless touch screen, confirmed each step, and issued start, pause, or stop commands. After verification, the robotic arm controlled the drilling trajectory in a supervised autonomous mode according to the preoperative plan under continuous operator supervision. Safety measures included real‐time optical tracking, visual display of the drill relative to the planned trajectory, stepwise verification before drilling, and immediate interruption of the procedure if tracking instability, system warning, excessive deviation, or unexpected patient movement occurred. No additional user‐defined numerical safety threshold was applied beyond the manufacturer′s built‐in safety monitoring workflow.

After routine disinfection and draping, the three‐dimensional printed positioning guide was checked intraorally for complete seating and stability. Patient registration was then performed using the positioning guide and the optical tracking system. The robotic arm, end effector, and implant handpiece were calibrated according to the manufacturer′s protocol. Before each osteotomy, the planned implant site, entry point, drilling depth, and angulation were verified on the software interface. After confirmation of tracking stability and trajectory consistency, the robotic system executed the osteotomy along the preoperatively planned path in a shared‐control manner under continuous supervision by the surgeon. The surgeon maintained control of irrigation, soft‐tissue protection, and procedure interruption throughout the surgery. At Site 15, a Straumann BL titanium implant, 4.1 mm in diameter and 10 mm in length, was placed directly against the base of the sinus septum. At Site 17, a Straumann BL titanium implant, 4.8 mm in diameter and 12 mm in length, was inserted with distal inclination along the posterior wall of the maxillary sinus. (Figure 5). Immediate postoperative CBCT imaging showed optimal implant position and angulation, in complete accordance with the preoperative plan (Figure 6). Postoperative CBCT data were matched and compared with preoperative design data to assess implant placement accuracy (Table 1). At Site 15, the entry point error was 0.434 mm, apex error was 0.577 mm, and angular deviation was 1.197°. At Site 17, the entry point error was 0.157 mm, apex error was 0.166 mm, and angular deviation was 1.084° (Figure 7), confirming high placement accuracy.

**Figure 5:**
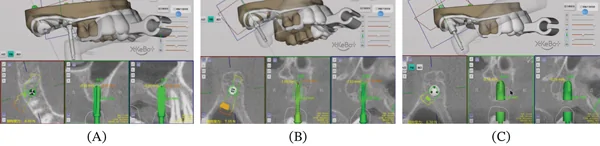
Autonomous implant robot prepares osteotomies and places implants according to the planned path. (A) Osteotomy preparation at 15. (B) Osteotomy preparation at 17. (C) Implant placement at 17. In the three panels (Aâ€“C) for the middle image, in the lower half of each, the left side is the lingual side, and the right side is the buccal side; in the right image in the lower half of each, the left side is the distal side and the right side is the mesial side.

**Figure 6:**
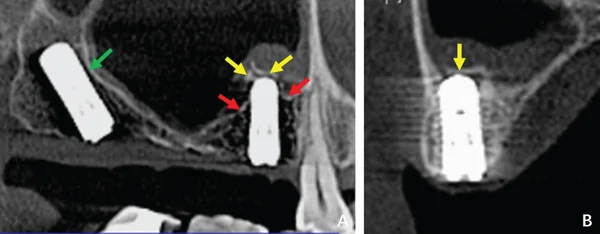
Immediate postoperative radiographic assessment at implant sites 15 and 17. (A) Sagittal view after surgery: The apex of the implant at 15 is precisely located at the base of the sinus septum. The yellow arrow indicates the sinus septum. The red arrows indicate the maxillary sinus floor at the mesial and distal aspects of the septum, demonstrating that the apex of the implant is within the septum but beyond the level of the adjacent sinus floor. The implant at 17 has been placed with distal inclination, closely adjacent to the posterior wall of the maxillary sinus, while maintaining a safe distance, as indicated by the green arrow. (B) Coronal view of the implant at 15: The apex of the implant is located within the bone of the sinus septum, as indicated by the yellow arrow.

**Table 1:** Accuracy analysis of autonomous robotic implant placement.

Implant site	Entry point deviation	Apex deviation	Angular deviation
15	0.434 mm	0.577 mm	1.197°
17	0.157 mm	0.166 mm	1.084°

**Figure 7:**
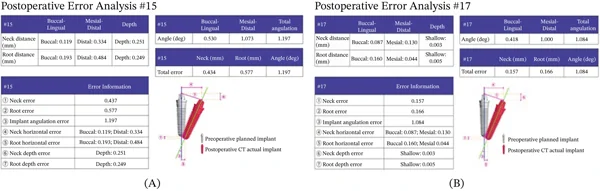
Analysis of implant placement accuracy. (A) Accuracy analysis of implant placement at 15. (B) Accuracy analysis of implant placement at 17.

Radiographic examination at 3 months postoperatively revealed no radiolucency around the implants and good osseointegration (Figure 8), after which the conventional second‐stage surgery was performed. Four weeks later, digital impressions were taken and an implant‐supported fixed prosthesis was fabricated (Figures 9 and 10). At the 15‐month follow‐up, radiographs showed that the implants remained in stable positions, with no areas of low bone density around them (Figure 11).

**Figure 8:**
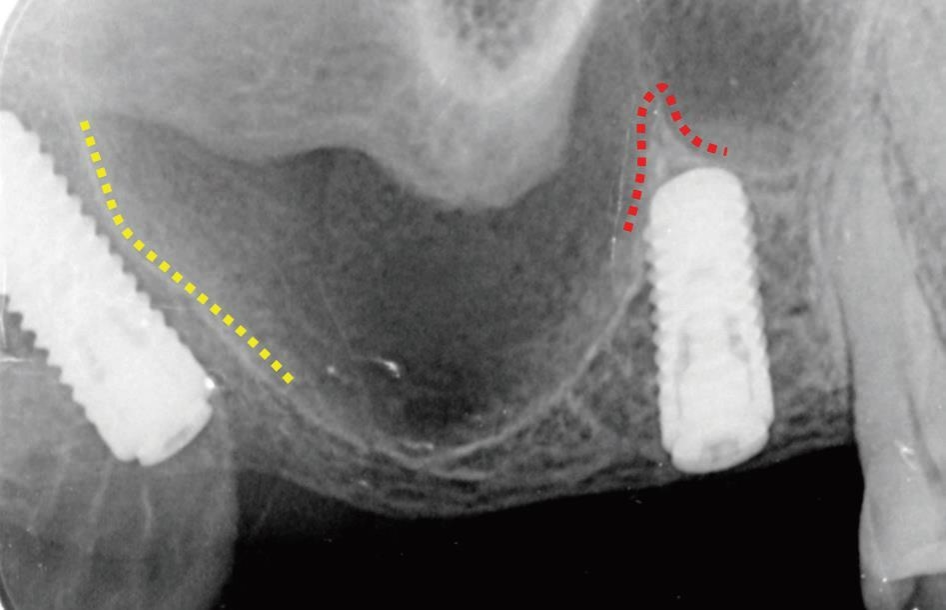
Radiograph at 3 months postoperatively shows good osseointegration. The red dashed line indicates the contour of the sinus septum, demonstrating that the apex of the implant at 15 is precisely located at the base of the septum; the yellow dashed line indicates the contour of the posterior wall of the maxillary sinus, showing that the implant at 17 is closely adjacent to the posterior wall while maintaining a safe distance.

**Figure 9:**
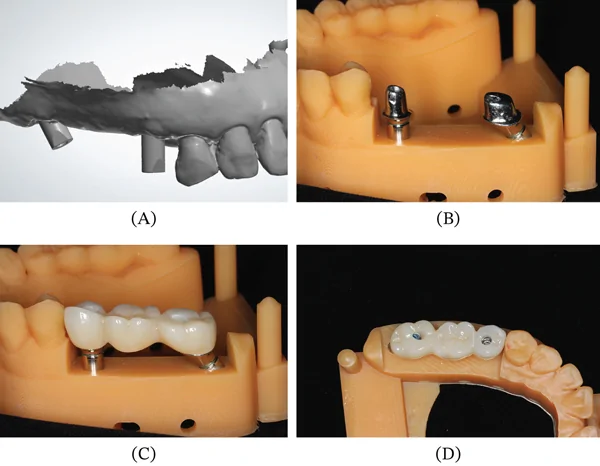
Digital fabrication of the definitive prostheses. (A) Digital impression. (B) Customized abutment. (C) Buccal view of the prostheses. (D) Occlusal view of the prostheses.

**Figure 10:**
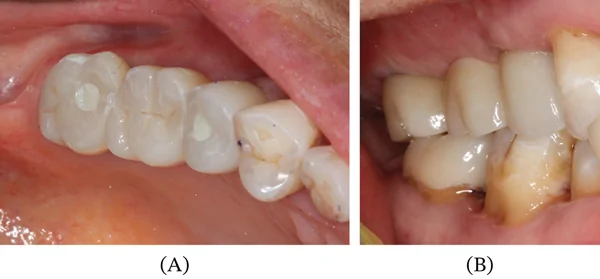
Delivery of the prostheses. (A) Occlusal view of prostheses, showing ideal screw access opening. (B) Buccal view of the prostheses.

**Figure 11:**
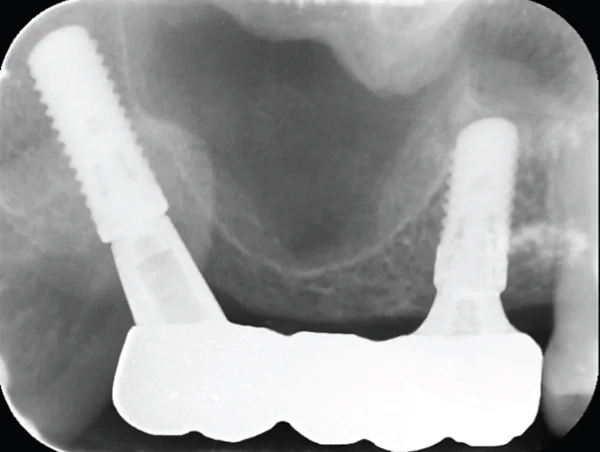
Radiograph at 15 months postoperatively shows good osseointegration with no marginal bone loss.

## 3. Discussion

After the loss of posterior maxillary teeth, factors such as pre‐existing periodontitis, alveolar bone resorption following extraction, and maxillary sinus pneumatization often lead to insufficient sinus‐crest distance. The conventional approach involves sinus floor elevation and/or alveolar ridge augmentation procedures to reconstruct adequate bone volume before carrying out implant placement, which typically extends the treatment period to 6–8 months and is associated with a range of potential complications, such as intraoperative sinus membrane perforation, postoperative sinus infection, graft exposure, or implant migration into the sinus cavity [4–7]. When local anatomical abnormalities are present, the difficulty and risk of surgery are further increased, for example, when large blood vessels are found in the lateral sinus wall, space‐occupying lesions are present in the sinus, or sinus septa exist [8–11].

In such situations, fixed restorations supported by both tilted and axial implants have become a reliable alternative. This approach enables implants to be placed within the existing bone, allowing for improved osseointegration and better prosthetic support. Tilted implant placement may also provide additional advantages, such as the possibility to use longer implants, which increases the implant‐to‐bone contact area and enhances primary stability. Furthermore, tilting implants increases the distance between neighboring fixtures, helping to optimize biomechanical distribution and avoid long cantilevers. Therefore, the combined use of tilted and axial implants offers the triple benefits of superior bone integration, a larger bone‐implant contact area, and enhanced biomechanical distribution. Some studies have also shown that tilted implants are subjected to greater bending moments at the abutment level; however, a rigidly connected fixed prosthesis can effectively distribute the stress within safe limits for both the implant and surrounding bone [12–15].

One study involving 3987 implants (2036 axial and 1951 tilted) found mean survival rates of 96.66% for axial implants and 96.93% for tilted implants, with no statistically significant difference. After 5 years of loading, marginal bone loss around tilted implants was 0.133 mm greater than that observed around axial implants, which was not statistically significant. Another study with 5–22 years of follow‐up also demonstrated similar implant survival and overall prosthetic success rates for both axial and tilted implants in full‐arch and partial fixed restorations, with no significant differences in marginal bone loss [16].

Accordingly, when posterior bone volume is insufficient and complex augmentation procedures are inadvisable (e.g., in elderly patients and those with large vessels within the sinus wall, or in patients with coexisting cardiovascular disease), or when a safer, less invasive, and shorter treatment protocol can be adopted, tilted implants may serve as an effective option for implant‐supported fixed restorations. However, to ensure optimal prosthetic outcomes, it is essential to leverage digital technologies—including CBCT, intraoral scanning, and three‐dimensional reconstruction—to facilitate prosthesis‐driven designs, assess the relationship between implant emergence and prosthesis, evaluate the safety margins relative to the maxillary sinus and adjacent teeth, and analyze spacing between implants.

In the present case, both the bone height and width at Sites 16 and 17 were insufficient, with concomitant diffuse mucosal thickening of the sinus. With a traditional approach, it would be necessary to first treat the sinus inflammation, followed by sinus floor elevation and horizontal bone augmentation, resulting in a longer and more invasive procedure. Furthermore, this conventional approach would require the complex selection of an appropriate grafting biomaterial. Previous comparative studies have demonstrated that various biomaterials used in sinus augmentation, such as autologous bone, calcium sulfate, and deproteinized bovine bone, exhibit significantly distinct resorption profiles and osteoconductive properties [17], adding variability and unpredictability to the healing process and final bone quality. By contrast, under the principles of prosthetically guided implantology and through the adoption of digital technologies, this case simulated the definitive prosthetic position and occlusion, then used this information in combination with local anatomical features to plan a distally tilted implant at Site 17 to avoid the sinus. At Site 15, the presence of a sinus septum with a broad base provided sufficient bone support for implant placement and obviated the need for sinus floor elevation. Three months after surgery, osseointegration was confirmed and definitive restoration was carried out, whereas with conventional sinus floor elevation a healing period of 6–8 months would have been required. Thus, the treatment period was shortened by 3–5 months. In addition, root‐form implants were selected whenever possible to avoid perforating the base of the septum. Preoperative simulation indicated that the emergence of the implant corresponded closely with the central fossa of the prosthesis, resulting in an optimal outcome. Even if local anatomical constraints prevent the emergence from being ideally positioned, or a short cantilever is required, as long as biomechanical and biological principles are respected, this strategy can decrease procedural complexity and reduce patient trauma while maintaining clinical effectiveness.

During impression taking, mutual interference between impression copings may occur, making it impossible to insert the copings simultaneously—particularly in cases where the first and second molars are missing, with an axially or mesially tilted implant at the first molar site and a distally tilted implant at the second molar site. In such situations, the following sequential method can be implemented: Insert and scan the coping for one implant, remove it, then insert and scan the coping for the other implant, and finally match the datasets, to obtain a complete impression for all implants.

With recent advances in artificial intelligence in dentistry and the high precision demonstrated by robotic implant placement, this case utilized an autonomous robotic system for surgery. Postoperative accuracy analysis confirmed that the robot achieved highly precise and efficient placement in accordance with the preoperative plan. Although there are a few reports of robotic implant surgery being used for sinus floor elevation, these primarily focus on the robot′s precision in improving the safety of the procedure and reducing complications such as membrane perforation [18–20]. However, the use of robotic assistance to perform tilted implant placement while avoiding the sinus—particularly leveraging the sinus septum base for support and thereby eliminating the need for sinus floor elevation, as in this case—represents an emerging clinical approach that further expands the indications for automated surgical execution.

Static computer‐assisted implant surgery, dynamic navigation, semiautonomous/navigation‐based robotic systems, and autonomous robotic systems represent different levels of digital assistance in implant dentistry. Static surgical guides are relatively simple, economical, and clinically established; however, they do not allow real‐time intraoperative adjustment, and their accuracy may be affected by guide fit, sleeve tolerance, drill length, mouth opening limitation, and accumulated manufacturing errors. Dynamic navigation provides real‐time visualization of the drill position and allows intraoperative modification of the plan, but the final accuracy remains highly dependent on the surgeon′s hand stability, visual attention, and ability to coordinate the navigation display with manual drilling. Although some comparative studies and systematic reviews have noted that robotic‐assisted implant placement may offer favorable control over angular deviation compared with static or dynamic guided surgery [21], semiautonomous or navigation‐based robotic systems may enhance positional control and reduce hand tremor, but many still require the surgeon to manually guide the robotic arm or execute drilling movements. In contrast, supervised autonomous robotic systems can execute the planned osteotomy trajectory through a mechanically stable arm after registration and calibration, potentially reducing operator‐dependent deviations caused by fatigue, tremor, limited visibility, or unfavorable posture, whereas the surgeon retains ultimate control over critical intraoperative management such as irrigation and soft‐tissue protection. Despite these potential mechanistic advantages, it should be emphasized that similar overall clinical outcomes in terms of implant positioning accuracy can also be achieved through careful digital planning combined with either static or dynamic guided implant surgery in experienced hands. Consequently, the primary advantage of robotic technology does not necessarily lie in an absolute increase in clinical accuracy, which can already be achieved with contemporary digital workflows, but rather in the automation of the surgical procedure, the standardization of treatment protocols, and the reduction of operator dependency.

In the present case, the surgical target was particularly demanding because the apical portion of the implant at Site 15 had to be positioned within the limited basal bone of the sinus septum while avoiding deviation toward the lower sinus floor on either side. Similarly, the implant at Site 17 required distal inclination along the posterior wall of the maxillary sinus while maintaining a safe distance from the sinus cavity. These requirements made accurate control of both entry point and angular trajectory critical. Therefore, autonomous robotic placement was selected not merely to achieve high accuracy, which is also attainable with other contemporary digital workflows, but primarily to automate the surgical execution, standardize the drilling process, and reduce the risk of sinus perforation by minimizing operator‐dependent variables. Nevertheless, autonomous robotic systems also have limitations, including cost, equipment size, workflow complexity, the need for calibration and registration, dependence on stable optical tracking, and the requirement for specific operator training [22].

Robot‐assisted implant surgery does not require clinicians to have highly advanced surgical skills, but rather necessitates systematic training in robot operation [23]. Nevertheless, there is substantial potential for further improvement. For example, the optical tracking systems currently used by robots can be affected by variations in lighting within the operating room, introducing error [24]. The presence of blood and saliva, limited mouth opening, patient head and tongue movement, and differences in bone density can also impact accuracy. Additionally, robots currently require preoperative calibration and re‐calibration if reference markers are disturbed during surgery, adding procedural complexity.

Given that dental robotics is still in its nascent stage and current systems are costly, technically complex, and physically large with limited intelligence, the clinical use of robots in implant dentistry remains somewhat restricted. However, as technology advances, robotic systems are expected to become more cost‐effective, accessible, and user‐friendly, achieving broader clinical application. Future developments in visual tracking, toward systems approximating human vision capable of recognizing adjacent anatomical structures (such as neighboring teeth, opposing teeth, and the edentulous ridge) and integrating with surgical planning may further obviate the need for physical reference markers and calibration devices.

In summary, for cases of insufficient sinus‐crest distance in the posterior maxilla, the combination of precise digital planning, comprehensive simulation, and prosthesis‐driven restoration utilizing both tilted and axial implants offers a reliable and efficient clinical solution, provided that anatomical conditions are suitable. However, it must be acknowledged that the inherent limitation of a single case report is that these findings cannot be widely generalized. Well‐designed clinical studies with larger sample sizes are necessary to confirm these observed outcomes and to establish definitive clinical guidelines.

## 4. Conclusions

In this case, a patient with insufficient bone height in the posterior maxilla successfully received fixed implant restoration utilizing digital planning and autonomous robotic‐assisted placement of tilted and axial implants. The advantages of this approach include minimizing surgical trauma, reducing treatment time and risk, improving implant placement accuracy, and achieving predictable clinical outcomes, particularly in anatomically challenging situations where conventional augmentation procedures may be invasive or contraindicated.

## Author Contributions

R.X. and Y.F. contributed equally to this work as co‐first authors. R.X. contributed to the robotic surgical planning and manuscript drafting. Y.F. participated in the clinical data collection and case analysis. R.X. performed the robotic‐assisted implant placement and postoperative evaluation. P.Z. and X.Y. contributed to the CBCT imaging analysis and anatomical measurements. J.D. supervised the prosthetic rehabilitation and follow‐up assessments. Y.Z. and Z.F. served as co‐corresponding authors, with Y.Z. overseeing the research design and Z.F. providing critical revisions to the manuscript. R.X. and Y.F. contributed equally to this article.

## Funding

This research was supported by New Technology Project (Shaanxi, China) (LX2021‐406) and National Clinical Research Center for Oral Diseases (Shaanxi, China) (LCB202403).

## Disclosure

All authors have read and approved the final manuscript.

## Ethics Statement

Informed consent about clinical management was obtained from the patient in this case. Written informed consent for publication of their details was obtained from the patient and was compliant with the hospital.

## Consent

A written informed consent for publication was obtained from the patient to publish all clinical data and any accompanying images, and also a written consent to publish this information was obtained from study participant.

## Conflicts of Interest

The authors declare no conflicts of interest.

## Data Availability

The datasets used and/or analyzed during the current study available from the corresponding authors on reasonable request.
